# Seroprevalence and Associated Risk Factors of Human Brucellosis in a Farming and Animal Health Community in South Africa, 2015–2016

**DOI:** 10.3390/tropicalmed10110302

**Published:** 2025-10-23

**Authors:** Jennifer Rossouw, Anastasia N. Trataris-Rebisz, Stefano Tempia, Melinda K. Rostal, William B. Karesh, Veerle Msimang

**Affiliations:** 1 Centre for Emerging Zoonotic and Parasitic Diseases, National Institute for Communicable Diseases, National Health Laboratory Service, Johannesburg 2192, South Africa; veerlem@nicd.ac.za; 2 Division for Biosafety and Biosecurity, National Institute for Communicable Diseases, National Health Laboratory Service, Johannesburg 2192, South Africa; anastasian@nicd.ac.za; 3 Partnership for International Vaccine Initiative, Tkforce for Global Health, Atlanta, GA 30329, USA; tempiastefano903@gmail.com; 4 One Health Research Consulting, Glen Rock, NJ 07452, USA; rostal@onehealthresearch.net; 5 One Health Concepts, Easton, CT 06612, USA; wbk@onehealthconcepts.com

**Keywords:** *Brucella*, brucellosis, seroprevalence, farming community, veterinary professionals, risk factors, South Africa

## Abstract

Brucellosis is a widespread zoonotic disease and a major contributor to febrile illness, often underdiagnosed. This study investigated the seroprevalence of brucellosis and the associated exposure factors within South African farming and animal health communities. A cross-sectional survey was conducted across 40,000 km^2^ in the Free State and Northern Cape provinces from 2015 to 2016. Interviews and serum samples were collected from 847 volunteers on randomly selected farms and veterinary professionals listed in a regional register. Samples were tested using a commercial *Brucella* IgG ELISA. Risk factors were assessed using logistic regression, accounting for within-farm clustering. The seroprevalence was higher among veterinary professionals (11.6%; 16/138) than farm-based participants (7.0%; 50/711); *p* = 0.095. Multivariable analysis identified several exposure factors within the farm-based population: age over 40 years (aOR = 5.35; 95% CI: 1.68–17.02), White population group (aOR = 4.60; 95% CI: 1.64–12.91), contact with diseased animals (aOR = 2.01; 95% CI: 1.05–3.84), and working 4–8 h daily with ungulates (aOR = 2.90; 95% CI: 1.25–6.76). Among veterinary professionals, odds of exposure were higher with more than 5 years of work (OR = 1.82; 95% CI: 1.21–2.72) and extended ungulate contact (OR = 4.85; 95% CI: 1.27–18.52). Occupational exposure highlights the need for improved brucellosis prevention strategies.

## 1. Introduction

Brucellosis is an important zoonotic disease that affects both livestock and humans, creating considerable public health challenges worldwide. Although it is often reported that 500,000 new human cases occur annually, this figure greatly underestimates the true global burden [1]. This underestimation stems largely from inadequate diagnostic infrastructure in high-burden countries and the clinical similarity of brucellosis to other febrile illnesses. A recent study suggests the true annual incidence may range from 1.6 and 2.1 million cases, with approximately 500,000 occurring in Africa alone [2]. This disease imposes a significant economic burden in affected regions due to livestock losses and the chronic health conditions it causes in some humans [3].

Brucellosis is a disease caused by bacteria of the genus *Brucella* that affects numerous animal species, including cattle, small ruminants, and pigs. It is transmitted to humans through direct contact with diseased animals, aborted foetuses, and infected animal tissue or by consuming contaminated animal products, such as undercooked meat, unpasteurized milk, or other dairy products [4]. Brucellosis poses an occupational risk to individuals working in the livestock sector, including farmers, butchers, veterinarians, and laboratory personnel, due to their frequent direct contact with animals or handling of potentially contaminated animal products [5]. In humans, brucellosis presents with a wide range of symptoms, from mild to severe, which can make diagnosis more tenuous due to frequent mimicry of other medical conditions. Common symptoms include intermittent fever, joint pain (arthralgia), muscle pain (myalgia), anorexia, gastrointestinal disturbances, spontaneous abortion, and profound fatigue. In some cases, individuals may develop neurological disorders. Collectively, these symptoms can lead to a significant decline in quality of life and progress to a chronically incapacitating disease with severe complications [5]. To effectively manage the condition and lessen its overall impact on health, it is imperative for diagnosis and treatment to be made early in the course of the disease. Human brucellosis has been treated with various antibiotics, either alone or in combination. However, monotherapy is not recommended due to a high relapse rate. When appropriate, a six-week course of doxycycline combined with streptomycin (administered for two to three weeks) is more effective and better tolerated than a six-week course of doxycycline with rifampicin [6]. However, cost and access to care are important considerations when selecting between these regimens, as the doxycycline–streptomycin combination requires daily intramuscular injections [6].

Human brucellosis was first recorded in southern Africa in 1894 in the then Orange Free State and Basutoland, with subsequent cases reported across the region [7]. The most recent official incidence data (0.1–0.3 cases per 100,000 population) dates back over four decades, based on notifications from 1977 to 1984 [8]. Despite being a notifiable condition, brucellosis remains underdiagnosed and underreported due to its nonspecific presentation, limited diagnostic capacity, and lack of clinical familiarity [9]. With no licensed human vaccine and rare person-to-person transmission, prevention depends on controlling the disease in animals, pasteurizing milk, and ensuring food hygiene. This underscores the need for a coordinated One Health approach.

Animal brucellosis is a controlled disease in South Africa, affecting both communal farming and intensive cattle production systems across all nine provinces, with higher prevalence in the central and Highveld regions [10]. Widespread outbreaks pose risks to livestock and human health, and the disease causes significant economic losses in the dairy and beef industries due to reproductive failure and reduced productivity [11]. A review of the country’s brucellosis control measures was conducted to strengthen collaboration between government, industry, and farmers [12]. Veterinary control measures include mandatory vaccination of heifers (4–8 months) with *B. abortus* Strain 19 or RB51, targeted testing of high-risk herds, movement restrictions for infected animals, and test-and-slaughter policies. However, low compliance with vaccination and testing, along with poor fencing, communal grazing, and lack of traceability systems, continues to drive transmission [12,13].

In recent years, there has been growing interest in human brucellosis in South Africa, with several studies exploring its epidemiology, risk factors, and public awareness [13,14,15,16,17,18]. These studies have highlighted poor knowledge and high-risk practices among populations such as communal cattle keepers and abattoir workers, underscoring the need for improved education and biosecurity measures [13,17]. Despite the increase in research, significant gaps remain in the understanding of the epidemiology of brucellosis in South Africa. Although previous studies have provided valuable insights into *Brucella* seroprevalence, many have been reliant upon modest to moderate sample sizes (e.g., 74 to 327 participants) [14,15,16,17,18]. A larger-scale study would improve statistical reliability, enabling more precise prevalence estimates in the study area, which is characterized by a substantial agricultural industry. Expanding research efforts would contribute to a more comprehensive understanding of human brucellosis in South Africa.

This study aimed to investigate the seroprevalence and exposure factors associated with human brucellosis in a farming and animal health community in central South Africa. The findings will support the development of targeted interventions to reduce zoonotic transmission and improve public health outcomes in the region.

## 2. Materials and Methods

### 2.1. Study Design and Setting

**Figure 1:**
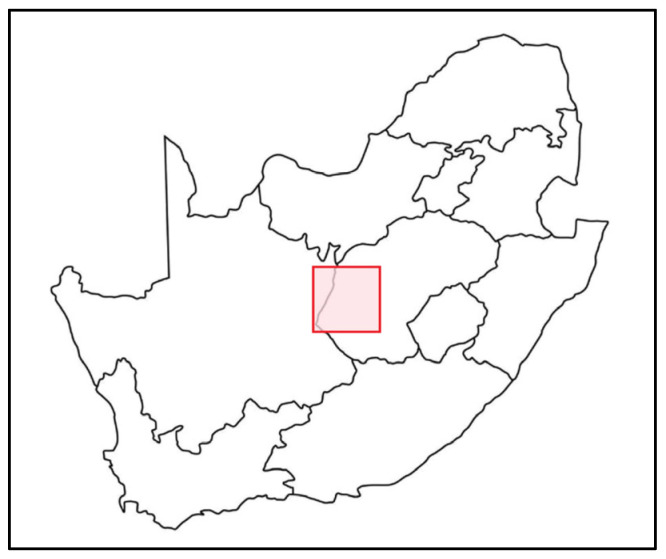
Map of South Africa showing the 40,000 km^2^ study area (red square) located within the Free State and Northern Cape provinces.

The data used in this study was collected during a sero-epidemiological survey conducted as part of a broader project investigating the presence of antibodies against a panel of zoonotic pathogens among various at-risk occupational groups. Within this larger study, a cross-sectional survey took place between October 2015 and February 2016. Due to the focus on Rift Valley fever (RVF), the research project concentrated on a defined 40,000 km^2^ (200 km × 200 km) area that included widespread ruminant livestock farming communities of the Free State Province and a small part of the Northern Cape Province, which had previously experienced large RVF outbreaks (Figure 1). This region features diverse farm sizes and production systems, with varying degrees of human–livestock interaction, all of which align with established epidemiological risk factors for *Brucella* transmission. Importantly, this area has a documented history of bovine brucellosis. Outbreaks reported to the Department of Agriculture, Forestry and Fisheries between 2010 and 2014 (Figure S1) indicate multiple confirmed outbreaks in the five-year period preceding the study. This historical burden of animal infection further supports the epidemiological relevance of the selected region for investigating human brucellosis seroprevalence.

The procedure for farm selection was previously described by Msimang et al. [19]. Briefly, in the absence of a comprehensive list of farms, random geographic coordinates were generated within the study area. The probability of selection was proportional to the density of livestock-owning households, based on data from Statistics South Africa. Farms were invited to participate in the survey in order of geographic proximity to the random geographic coordinates. For veterinary professionals working in the study area, a complete sampling list was obtained from the South African Veterinary Council register.

The study was conducted within a well-defined farming community comprising approximately 40,000 individuals, alongside 200 registered veterinary professionals working across multiple veterinary facilities. A sample size of 770 individuals was estimated for a maximum *Brucella* seroprevalence of 50%, with a 95% confidence interval (CI), 5% precision, 0.2 intra-cluster correlation, 2 design effect, and an average cluster (farm) size of 6 [20]. All veterinary practitioners and associated professionals were invited to enroll; as such, no sample size estimate was conducted.

### 2.2. Recruitment

At the farms, all people living on the farm and/or working with livestock were invited to participate, and all consenting target individuals on the selected farms were enrolled. All veterinary practitioners, as well as paraveterinary and assisting staff of facilities registered with the South African Veterinary Council within the study area, were invited to participate in the study.

### 2.3. Data Collection

The farm owner or manager provided written informed consent to conduct the survey on a day convenient for each establishment. They also responded to operational questions about the farm, such as the date of establishment, numbers/species/source of animals, equipment available, and hygiene procedures in place. This information was captured in a pretested, structured questionnaire on a tablet using the Open Data Kit (ODK) application.

The study team explained the purpose of the study to the farming population (hereafter also referred to as farm-based participants) as well as to veterinarians, paraveterinarians, and associated support staff (hereafter collectively referred to as veterinary professionals). After obtaining written informed consent, a blood sample was collected. Participants, assisted by a study team interviewer, completed a piloted, structured questionnaire on a tablet using the ODK application. The questionnaire included questions regarding age, sex, education, job description in the workplace, exposure to animals and meat carcasses, and activities of workers on the farm or veterinary professionals. The questionnaire also covered known risk factors for both RVF and brucellosis, such as eating raw meat, consuming unpasteurised milk (or fresh unboiled milk), consuming dairy products prepared from unboiled milk, preparing dairy products at home, handling animals, animals’ vaccination status, handling animal tissues (e.g., placenta, aborted foetus, and birth fIuids), and slaughtering/skinning animals within 12 months from the date of completing the questionnaire, whether the livestock on the farm were vaccinated against *Brucella* or whether they experienced a brucellosis outbreak in the past. Additionally, it asked about brucellosis-related symptoms, healthcare-seeking behaviour, use of personal protective equipment, chronic conditions, and treatment history.

### 2.4. Laboratory Testing

Blood samples (collected in serum separator tubes) were centrifuged at 3000 rpm and stored in a fridge (4 °C) until transport to the National Institute for Communicable Diseases. Upon receipt at the laboratory, serum samples were promptly divided into aliquots and stored at −20 °C until processing. Serum samples were tested using the Vircell *Brucella* IgG enzyme-linked immunosorbent assay (ELISA) kit (Vircell S.L., Granada, Spain), following the manufacturer’s instructions [21]. The Vircell *Brucella* IgG ELISA was selected for its suitability in detecting past exposure, high-throughput capacity, and alignment with national public health diagnostic protocols. According to manufacturer data [21], the assay demonstrates a sensitivity of 98% (95% CI: 89–100%) and a specificity of 100% (95% CI: 94–100%), supporting its reliability for seroprevalence studies.

### 2.5. Data Analysis

Data analysis was conducted using Stata version 13 (Quantec, StataCorp LLC, College Station, TX, USA). Descriptive statistics were used to summarise the data, with percentages calculated for *Brucella* antibody prevalence. To account for the clustered cross-sectional survey design in farms, 95% confidence intervals (CI) were constructed using the linearized variance estimator based on a first-order Taylor series linear approximation. Logistic regression was used to assess the association between potential risk factors and the apparent *Brucella* serological status of study participants. For the multivariable logistic regression model, all variables with a *p*-value < 0.2 on univariable analysis were included. Non-significant factors (*p* > 0.05) were then dropped using manual backward elimination. The job description variable for each occupation was included in the model regardless of its significance to control for by multivariable adjustment for potential confounding effects, which could produce spurious associations if unaccounted for. Analysis was conducted with adjustment for data collected using a survey sampling design and clustering. The svy-set command specified the farm identifier as the primary sampling unit (cluster) variable, and the svy-prefix was used for estimation and risk factor analysis commands. The model for veterinary professionals was fit by penalized maximum likelihood regression. The geographical coordinates of the workplaces where participants worked or lived were used to map the spatial distribution of the proportion of seropositive individuals at each workplace using ArcGIS Pro 2.3.0 (Esri, Redlands, CA, USA).

## 3. Results

### 3.1. Characteristics of Farm Workplace and Study Participants

This section presents descriptive statistics for the study population, providing context for the inferential analyses presented in Section 3.3. Of the 849 individuals tested, 711 (83.7%) were farm-based participants recruited from 209 farms, while 138 (16.3%) were veterinary professionals. Of the farm participants, 695 worked on domestic animal farms (n = 204), whereas 16 participants worked on farms that primarily kept game animals (n = 5). The median age of participants with available data was 36 years (range: 16–84 years) for farm-based individuals, and 37 years (range: 22–73 years) for veterinary professionals.

Among farm-based participants, the proportion of males was 92.5% (95% CI: 90.4–94.4), which was much higher than that of females at 7.5% (95% CI: 5.6–9.6). Among veterinary professionals, the sex distribution was more balanced with 49.3% males (95% CI: 40.7–57.9) and 50.7% females (95% CI: 42.1–59.3).

Of the domestic animal farms, 93.7% of participants (666/711) were sampled from 184 farms located on private land, while 6.3% (45/711) were sampled from 20 farms situated on communal land. On private land farms, the median number of participants enrolled was 3 (range: 1–14), out of a median of 4 employed individuals (range: 0–45). In contrast, communal land farms had a median of 2 participants (range: 1–8) out of 2 employed individuals (range: 0–35). The median size of privately owned farms ranged from 1001 to 2000 hectares.

Regarding occupational roles, 71.0% of farm-based participants (505/711) were employed as farm labourers or herdsmen (including several hired wool shearers), 25.2% (179/711) were managers or owners of farms or livestock, and 3.8% (27/711) were family members, housewives, domestic helpers, or drivers. Among the veterinary professionals, 48.6% (67/138) were veterinarians, 32.6% (45/138) were veterinary technicians, animal health technicians, or para-veterinarians, and the remaining 18.8% (26/138) included veterinary nurses, veterinary laboratory technologists, researchers, conservation workers, and veterinarians practicing farming. The sample represented 35% (69/200) of the registered veterinarians in the study area.

### 3.2. Seroprevalence Estimates

**Figure 2:**
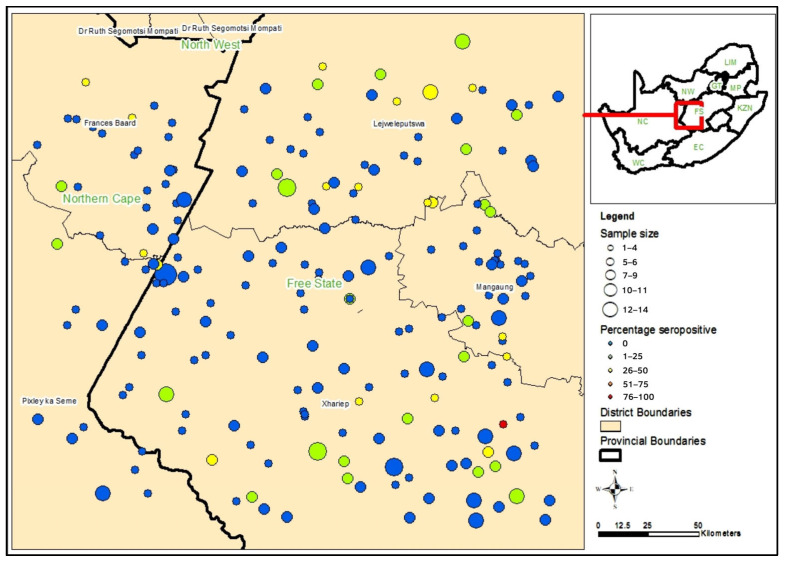
Spatial distribution of *Brucella* seropositivity in a farming and animal health community in the Free State and Northern Cape provinces, 2015–2016.

A total of 7.8% (95% CI: 6.1–9.9%) of participants (66/849) exhibited IgG seroreactivity to *Brucella* spp. The analysis revealed a seroprevalence point estimate of 11.6% (16/138; 95% CI: 6.8–19.0%) among veterinary professionals and 7.0% (50/711; 95% CI: 5.3–9.2%) among the farm-based population with adjustment for clustering in this group. Veterinary professionals had 1.73 (95% CI: 0.91–3.31) times greater odds of being seropositive for brucellosis (*p* = 0.095) compared to the farm-based group. Figure 2 illustrates the spatial distribution of *Brucella* IgG seropositive participants across the study area.

**Figure 3:**
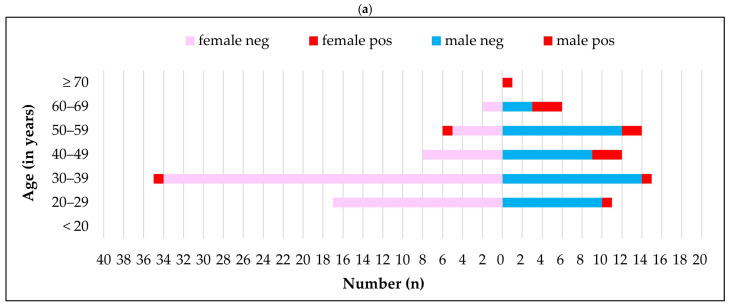

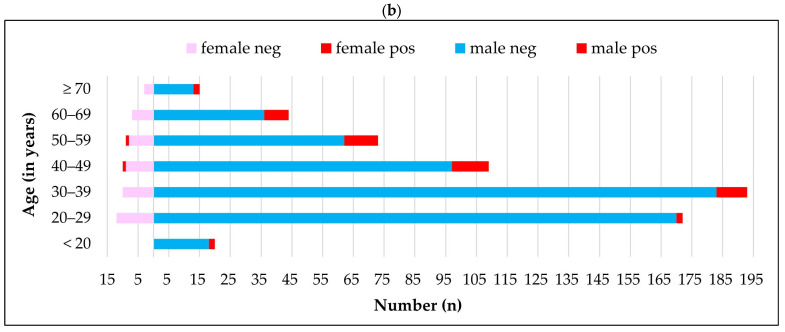
Age–sex distribution of *Brucella* IgG seronegative and seropositive participants shown in two panels: (**a**) veterinary professionals and (**b**) farming population in the Free State and Northern Cape provinces, 2015–2016.

The number of male and female farm-based participants varied significantly, but the difference in seropositivity between sexes was not statistically significant, with 48 of 658 males (7.3%) and 2 of 53 females (3.8%) testing seropositive (*p* = 0.133). In contrast, among veterinary professionals, seropositivity for brucellosis differed significantly by sex: 20.6% of males (14/68) were seropositive compared to 2.9% of females (2/70) (*p* < 0.007; Table 1 and Table 2; Figure 3). Males in both groups had seropositive individuals across all age groups sampled, whereas seropositive females were observed exclusively in the 39–59 age band.

**Figure 4:**
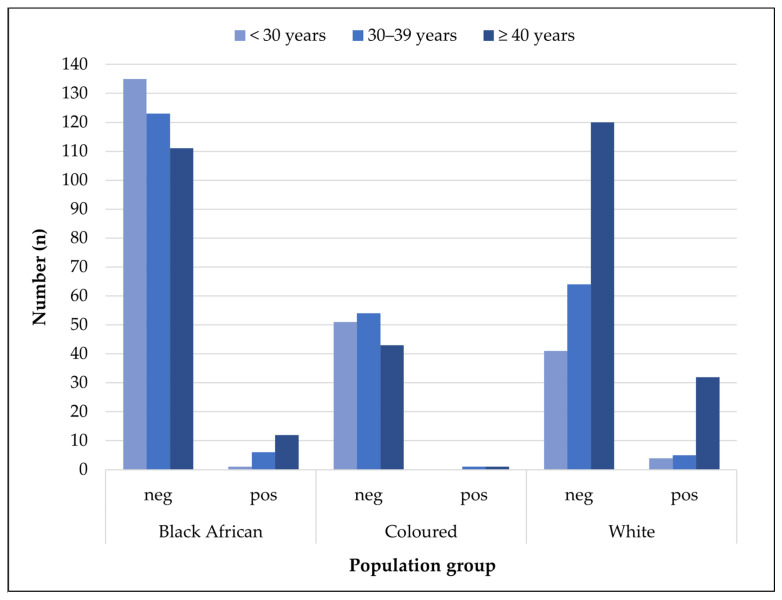
Age group trends in *Brucella* IgG seronegative and seropositive participants, categorized by population group, in a farming and animal health community in the Free State and Northern Cape provinces, 2015–2016.

Across both farm-based and animal health groups with available age and population data, seropositivity was notably higher among White participants, with 15.4% (41/266) testing positive. This trend was especially pronounced in individuals over 40 years old, where 32 of the 152 seropositive cases occurred among White participants (Figure 4). In contrast, Black African participants had a lower seropositivity rate of 4.9% (19/388), while Coloured (mixed race) participants showed the lowest rate at 1.3% (2/150).

### 3.3. Multivariable Analysis of Risk Factors Associated with Brucella IgG Seropositivity

**Figure 5:**
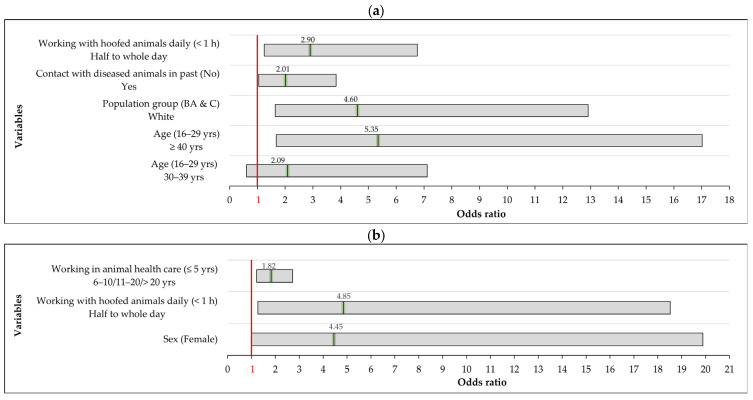
Factors associated with *Brucella* IgG seropositivity, along with their corresponding (adjusted) odds ratios (aOR/OR) (green) and 95% confidence intervals (black boxes) for significant variables in the final logistic regression model. Results are presented for: (**a**) Farm-based population and (**b**) Veterinarians and associated professions in the Free State and Northern Cape study area. The red line represents an OR of 1. BA = Black African, C = Coloured (mixed race) population.

To initiate the logistic regression analysis, all 30 variables with a *p*-value < 0.20 from the exploratory univariable analysis of the farm-based population were included in the initial model. The final multivariable analysis identified several risk factors that were significantly associated with *Brucella* seropositivity within this population (Table 1). After adjusting for other variables in the multivariable model, four factors emerged as significant predictors of *Brucella* seropositivity (Figure 5). Increasing age was associated with significantly higher odds of *Brucella* exposure: individuals aged 40 years or older had 5.35 times higher odds of exposure compared to those aged 16–29 years (aOR = 5.35; 95% CI: 1.68–17.02; *p* = 0.005). Additionally, members of the White farm-based population were 4.60 times more likely to show evidence of *Brucella* exposure than their Black African or Coloured (mixed race) counterparts (aOR = 4.60; 95% CI: 1.64–12.91; *p* = 0.004). Close contact with hoofed livestock or game animals for half a day (4 h) or a whole day (8 h) on a typical workday was also significantly associated with increased seropositivity, compared to contact of less than one hour (aOR = 2.90; 95% CI: 1.25–6.76; *p* = 0.014). Additionally, the study found that farm-based participants who reported having handled sick animals in the past had significantly higher odds of *Brucella* exposure compared to those who had not (aOR = 2.01; 95% CI: 1.05–3.84; *p* = 0.034).

Factors that were not significantly associated with *Brucella* seropositivity included assisting with abortions, involvement in slaughter or necropsy, history of brucellosis in farm animals, and vaccination status of livestock or game. Additionally, variables related to milk consumption and farm ownership type (private vs. communal land) were excluded from the multivariable model due to insufficient response counts (fewer than five in either category), which limited their statistical evaluation.

Likewise, several of the 14 variables that showed univariable associations with *Brucella* IgG seropositivity remained significantly associated in the multivariable analysis of veterinary professionals (Table 2). The multivariable analysis identified the following risk factors associated with a higher odds of *Brucella* IgG seropositivity in this group (Figure 5). Male participants had higher odds of being seropositive than females (OR = 4.45; 95% CI: 1.00–19.89; *p =* 0.05). Working as a veterinary professional for more than 5 years, compared to five years or less, was a strong predictor of seropositivity (OR = 1.82; 95% CI: 1.21–2.72; *p* = 0.004). Additionally, veterinary professionals who handled hoofed livestock or game animals for half to a full day on a typical day had significantly higher odds of *Brucella* IgG seropositivity compared to those with less than one hour of contact (OR = 4.85; 95% CI: 1.27–18.52; *p* = 0.021). Although prior contact with confirmed *Brucella*-positive animals initially indicated elevated exposure risk, it was not statistically significant in the final model and was excluded (OR = 14.1; 95% CI: 0.77–258; *p* = 0.075). The presence of IgG antibodies was not significantly associated with assisting in cattle abortion events or prior exposure to confirmed *Brucella*-positive animals or wildlife. Additionally, the variable on assisting with animal births was excluded from the multivariable analysis due to a low response count (fewer than five in the “no” category), which prevented meaningful assessment.

### 3.4. Associations Between Brucella Seropositivity and Self-Reported Symptoms

While the primary aim of this study was to assess *Brucella* seroprevalence, we also explored associations with self-reported symptoms. The questionnaire included symptoms such as fever, sore joints, headache, tiredness, feeling unwell, and miscarriage (females). In univariable analyses (*p* < 0.2), *Brucella* seropositivity among veterinary professionals showed associations with fever (reported in the past 12 months and past 2 weeks), headache (past 12 months), and tiredness (past 12 months and past 2 weeks). Among farm-based professionals, an association was observed with headache (past 12 months). However, none of these associations reached statistical significance at *p* < 0.05. Given that symptoms are manifestations of infection rather than predictors, they were not included in the multivariable model. It is also important to note that all symptom data were self-reported, and many symptoms such as fever, tiredness, and headache are nonspecific and commonly reported across a range of illnesses. No association was found between *Brucella* seropositivity and miscarriage among women in the veterinary group, and the number of women in the farm-based group was too small to allow meaningful assessment.

## 4. Discussion

This study presents the seroprevalence of human brucellosis in a farming and animal health community that lives and works on livestock and wildlife farms in central South Africa. It also examines the risk factors associated with brucellosis seroprevalence among the farming population and veterinary professionals.

The study population was primarily composed of farm-based professionals (83.7%), with a smaller proportion of veterinary professionals (16.3%). Most participants were sampled from domestic animal farms, with a significant proportion residing or working on privately owned land. This distribution of participants highlights the diversity in farm sizes, production systems and human–livestock interactions prevalent in the study area, which are critical factors in understanding brucellosis transmission dynamics.

Studies have shown that *Brucella* seroprevalence in humans varies across different provinces in South Africa, with reported rates ranging from 1.4% in acute febrile patients to 10.7–20.9% in high-risk occupational groups, based on IgG ELISA testing [14,15,16,17,18]. The findings of this study confirm the presence of *Brucella* IgG antibodies within the farming community in central South Africa, with an overall seroprevalence of 7.8%. Veterinary professionals exhibited a higher seroprevalence of 11.6%, when compared with the 7.0% reported among farm-based participants. It is surmised that their increased risk could be attributed to frequent, direct contact with a greater number of potentially infected animals, contaminated materials, and livestock vaccine strains, encountered during their routine examinations and treatment of animals across multiple farms [22,23]. The *Brucella* IgG percentages detected in the professionals working with mixed domesticated (sheep, cattle, goats) and wild ruminants in our study area are significantly lower than the 20.9% (ELISA) reported among cattle farm workers and veterinary officials in Gauteng Province [15]. This difference may be attributed to a higher prevalence of bovine brucellosis relative to the other animal species, as well as a higher prevalence within the Gauteng Province, as evidenced by the high number of outbreak notifications reported to the Department of Agriculture [10]. Although the study survey included a question regarding past *Brucella* detection on farms, a key limitation is the lack of confirmed brucellosis status of the farms at the time of data collection. In contrast, the Gauteng study was conducted on 30 case farms and 11 control farms, where the brucellosis status had been verified [15]. The lack of confirmed farm-level infection status limits the ability to correlate human seroprevalence directly with local animal infection. This limitation is further compounded by the absence of an active bovine brucellosis surveillance program in the study area during the study period, which further restricts the ability to evaluate zoonotic risk and interpret human seroprevalence in relation to livestock infection dynamics.

*Brucella* seroprevalence in humans varies across sub-Saharan Africa, largely influenced by occupation, geographical distribution, and livestock exposure [24,25,26]. This variability is particularly evident when comparing communities based on their livelihoods and exposure pathways. Pastoralist and agro-pastoralist populations consistently demonstrate high seroprevalence due to frequent and close contact with animals, with percentages reaching 48.3% (ELISA) in Afar pastoral communities in Ethiopia and 54% (ELISA) among pastoralists in northern Kenya [27,28]. In contrast, lower seroprevalence is observed in more structured farming environments, particularly among smallholder farmers and dairy workers. For instance, in Nigeria, seroprevalence ranged from 0% (ELISA) to 0.83% (Rose-Bengal test; RBT), depending on the diagnostic method, while Eritrea recorded 1.2–1.4% (RBT with cELISA confirmation) [29,30]. Differences in diagnostic tools and survey design, and sampling may also contribute to variation in reported percentages across regions. Recent seroprevalence studies in at-risk occupational groups have demonstrated substantial variation in seroprevalence depending on the test used [15,17,29,31]. These patterns underscore the need for context-specific surveillance and control strategies that account for ecological, occupational, and methodological differences across the continent.

In this study, *Brucella* IgG seroprevalence was significantly influenced by demographic and occupation-related factors among both farm-based participants and veterinary professionals, as revealed by multivariable regression analysis. Among farm-based participants, four independent predictors remained significant after adjusting for covariates. Occupation-related exposure played a critical role, with those working half to a full day with hoofed animals (aOR = 2.90) and individuals with prior contact with diseased animals (aOR = 2.01) having increased odds of being seropositive. White individuals exhibited a higher risk (aOR = 4.60), while participants aged ≥40 years demonstrated an increased odds of exposure (aOR = 5.35). Currently, there is limited research on ethnicity/race (population groups) as an independent risk factor for *Brucella* exposure. In this study, the observed association lacks a definitive explanation. It may reflect more nuanced differences in occupational roles, behavioural practices, or environmental exposures not captured in the questionnaire. One possible contributing factor is age, as the White farm-based population in our sample tended to be older, and older age has been associated with cumulative exposure risk in the farm-based group. Both age and ethnic group/race were included in the multivariable model, implying that ethnic group/race was controlled for age and still had additional explanatory power for increased *Brucella* IgG odds. Given these considerations, further research is warranted to explore potential explanations, incorporate more detailed exposure assessment (including frequency and duration of contact), control for confounding variables, and assess whether this finding persists across different populations and settings.

Similarly, veterinary professionals exhibited distinct risk patterns, with prolonged occupational exposure emerging as the strongest independent predictor. Specifically, working for more than 5 years as an animal healthcare worker (OR = 1.82) and spending half to a full day with hoofed animals (OR = 4.85) were significant predictors. Additionally, male sex (OR = 4.45) was nearly significant, suggesting potential associations that warrant further investigation in a larger survey. The risk factors identified in this study align with findings from other research. A study on veterinarians in Palestine reported similar risk factors, including prolonged occupational exposure and advancing age [23]. This association may also be attributed to the higher odds of exposure over time, especially considering that *Brucella* IgG antibodies can persist for many years. A recent study demonstrated that *B. abortus* can survive intracellularly despite recommended antibiotic treatment, potentially contributing to relapse [32]. Notably, *Brucella* IgG antibodies may remain detectable long after treatment, even in patients who do not relapse. Among non-relapsing patients, 89% remained IgG positive 12 months post-therapy [33].

Interestingly, several factors were not significantly associated with *Brucella* IgG seropositivity. Among farm-based participants, variables such as job description (farmer/livestock owner vs. farmworker), years of working on a farm or with animals, consumption of raw milk, assisting with birthing or surgery, performing post-mortem examinations, slaughtering animals, sharp-object injuries, and prior farm history of brucellosis or vaccination against brucellosis were not predictors of/protective factors for *Brucella* IgG seropositivity. Among veterinarians and related professions, prior contact with *Brucella*-positive animals, needle stick injuries, assisting with surgery, touching aborted foetuses, and handwashing practices after animal contact had no significant association with *Brucella* IgG outcome in the multivariable regression. A systematic review and meta-analysis of occupational exposure to *Brucella* spp. found that, in addition to direct contact with secretions and excretions from potentially infected animals, veterinary professionals face significant risk due to activities inherent to their work. Notably, they are among the most exposed to live attenuated *Brucella* vaccines (e.g., Rev.1, S19, and RB51), which have been documented as sources of human infection through accidental exposure [22]. Similar risks may apply to farm-based participants, particularly those involved in animal vaccination or handling potentially infected livestock, although this was not reflected in the statistical associations observed in this study. These findings underscore the need for targeted preventive strategies aimed at high-risk occupational groups, with particular attention to exposure-related practices and demographic vulnerabilities that may contribute to *Brucella* transmission.

This study has several important limitations that should be considered. The time elapsed between data collection and publication may affect the direct applicability of prevalence estimates to current conditions. Nonetheless, the data serve as a valuable historical benchmark for understanding disease dynamics and informing future surveillance efforts. In addition, its cross-sectional design captures data at a single point in time, limiting the ability to infer causality. Although certain demographic and occupational factors were associated with *Brucella* IgG seropositivity, their direct involvement cannot be established. Although the sample size of the veterinary professions subgroup was small, it represented 35% of the registered veterinarians in the study area. Therefore, only seropositivity estimates were calculated for this group. The sample size for the farm-based participants was smaller than the initially calculated requirement. However, a recent study reported a *Brucella* seroprevalence of 21% [17]. Using this percentage, rather than the default maximum of 50% when prevalence is unknown, and applying the same parameters outlined in the methods, the recalculated required sample size was 510, which is below the actual sample of 711 farm-based participants. The study’s scope was also limited to a specific region in central South Africa and a defined farming-related population, which may not reflect broader occupational or geographic contexts. Inferences may not apply to the broader population or to farming communities outside the study area, where transmission dynamics could differ. Furthermore, key variables such as consumption of unpasteurised milk products, use of personal protective equipment, and animal contact were self-reported, raising the potential for recall bias and socially desirable responses that could affect the accuracy of exposure data. Although the IgG ELISA is a useful assay for detecting past exposure to *Brucella* due to the persistence of IgG antibodies, its diagnostic accuracy may be compromised by cross-reactivity, variability in host immune response, and technical factors such as sample degradation, potentially resulting in false-positive and false-negative results. False-positive results can result in an overestimation of true exposure rates. Additionally, the study relied on manufacturer-defined cut-off values for seropositivity, which were not adjusted to account for the endemicity of *Brucella* in the region. In endemic areas, detectable IgG typically indicates prior exposure to *Brucella*, which may not necessarily reflect active infection or clinical illness, an important consideration when interpreting seroprevalence data [34].

In conclusion, this study provides important insights into the seroprevalence and associated risk factors of brucellosis in central South Africa. The findings underscore the need for targeted interventions to mitigate zoonotic exposure, particularly among farming communities and veterinary professionals. Enhancing diagnostic capabilities in livestock, promoting occupational safety through increased awareness and training, and ensuring adherence to *Brucella* vaccination guidelines are critical components of an effective control strategy. The adoption of a One Health approach that integrates human, animal, and environmental health perspectives is essential for coordinated surveillance, prevention, and response efforts. Public health initiatives should prioritize education on preventive practices, improve access to personal protective equipment, ensure the availability of appropriate antibiotic treatment, and coordinate with animal health officials. Collectively, these measures can contribute to a substantial reduction in brucellosis transmission and support the broader goal of safeguarding public and animal health in endemic regions. Importantly, this study also highlights the need for region-specific diagnostic thresholds and longitudinal follow-up of seropositive individuals to better understand brucellosis in endemic populations. These findings provide essential baseline data to inform future surveillance and public health initiatives.

## Figures and Tables

**Table 1:** Risk factors examined for association with *Brucella* IgG seropositivity via univariable and multiple variable analysis among participants in the farming population within the Free State and Northern Cape provinces, 2015–2016.

Farming Population		Univariate Analysis		Multivariable Analysis	
Variables	*Brucella* IgG Seropositive n/N (%)	Odds Ratio (CI 95%)	*p*-Value (* < 0.2)	Adjusted Odds Ratio (CI 95%)	*p*-Value (** < 0.05)
Sex					
Female	2/53 (3.8%)	1 (base)	-	1 (base)	-
Male	48/658 (7.3%)	2.73 (0.73–10.17)	0.133 *	2.12 (0.48–9.30)	0.317
Population group					
Coloured or Black African	21/536 (3.9%)	1 (base)	-	1 (base)	-
White	29/175 (16.6%)	5.11 (1.98–13.22)	0.001 *	4.60 (1.64–12.91)	0.004 **
Age (years)					
16–29	4/204 (2.0%)	1 (base)	-	1 (base)	-
30–39	10/203 (4.9%)	2.27 (0.66–7.81)	0.193 *	2.09 (0.61–7.12)	0.237
≥40	35/270 (13.0%)	5.21 (1.62–16.76)	0.006 *	5.35 (1.68–17.02)	0.005 **
Working on farm (years)					
≤5	10/320 (3.1%)	1 (base)	-	1 (base)	-
6–10	6/114 (5.3%)	1.53 (0.52–4.52)	0.440	1.18 (0.35–4.01)	0.791
11–20	13/139 (9.4%)	2.58 (0.99–6.70)	0.052 *	1.38 (0.45–4.28)	0.572
21–30	6/66 (9.1%)	2.11 (0.73–6.10)	0.165 *	1.00 (0.27–3.73)	0.997
31–40	3/39 (7.7%)	1.68 (0.44–6.48)	0.448	0.78 (0.18–3.44)	0.744
41–75	12/49 (24.5%)	5.60 (2.21–14.20)	0.000 *	1.56 (0.44–5.47)	0.486
Working with animals (years)					
≤5	7/258 (2.7%)	1 (base)	-	1 (base)	-
6–10	4/118 (3.4%)	1.12 (0.31–4.03)	0.862	0.85 (0.19–3.82)	0.831
11–20	16/156 (10.3%)	3.30 (1.35–8.08)	0.009 *	1.67 (0.48–5.79)	0.414
>20	23/192 (12.0%)	2.80 (1.16–6.72)	0.022 *	0.37 (0.06–2.34)	0.292
Job description					
Farmworker/herdsman	22/505 (4.4%)	1 (base)	-	1 (base)	-
Farmer/livestock owner; manager	27/179 (15.1%)	3.90 (2.21–6.88)	0.000 *	0.70 (0.26–1.23)	0.490
Other (family, domestic staff, driver)	1/27 (3.7%)	0.84 (0.11–6.65)	0.872	0.43 (0.05–3.84)	0.452
Help with transport of animals					
Yes	44/526 (8.7%)	2.04 (0.86–4.81)	0.103 *	0.85 (0.29–2.53)	0.769
No	6/185 (3.2%)	1 (base)	-	1 (base)	-
Cleaning of animal equipment					
Yes	46/573 (8.0%)	2.16 (0.77–6.05)	0.140 *	1.79 (0.63–5.11)	0.272
No	4/138 (2.9%)	1 (base)	-	1 (base)	-
Assisting with birthing of animals					
Yes	46/601 (7.7%)	2.00 (0.73–5.47)	0.177 *	1.04 (0.25–4.29)	0.916
No	4/110 (3.6%)	1 (base)	-	1 (base)	-
Assisting with surgery					
Yes	20/144 (13.9%)	1.88 (0.97–3.67)	0.062 *	1.17 (0.50–2.73)	0.712
No	30/567 (5.3%)	1 (base)	-	1 (base)	-
Slaughter animals					
Yes	44/585 (7.5%)	1.80 (0.76–4.28)	0.183 *	0.78 (0.24–2.53)	0.675
No	6/126 (4.8%)	1 (base)	-	1 (base)	-
Performing post-mortem					
Yes	23/159 (14.5%)	2.18 (1.11–4.27)	0.024 *	1.93 (0.91–4.1)	0.087
No	27/552 (4.9%)	1 (base)	-	1 (base)	-
Use of protective gear when working with animals					
Yes	9/254 (3.5%)	0.58 (0.27–1.25)	0.161 *	0.44 (0.18–1.09)	0.076
No	41/456 (9.0%)	1 (base)	-	1 (base)	-
Working with hoofed animals					
Half to whole day	44/595 (7.4%)	2.63 (1.12–6.16)	0.027 *	2.90 (1.25–6.76)	0.014 **
<1 h	6/116 (5.2%)	1 (base)	-	1 (base)	-
Contact with diseased animals in the past					
Yes	30/257 (11.7%)	2.32 (1.26–4.27)	0.007 *	2.01 (1.05–3.84)	0.034 **
No	20/470 (4.3%)	1 (base)	-	1 (base)	-
Suffered injury from sharp object					
Yes	26/251 (10.4%)	1.68 (0.94–2.99)	0.079 *	1.08 (0.51–2.31)	0.839
No	24/476 (5.0%)	1 (base)	-	1 (base)	-
Drink milk ^1^					
Yes	48/604 (7.9%)	4.19 (0.98–18.04)	0.054 *	-	-
No	2/107 (1.9%)	1 (base)	-	-	-
Drinking raw milk					
Yes	26/309 (8.4%)	2.09 (0.96–4.56)	0.064 *	1.53 (0.66–3.56)	0.320
Sometimes	7/58 (12.1%)	2.22 (0.85–5.76)	0.102 *	1.73 (0.51–5.83)	0.375
Always boiled or pasteurised	15/237 (6.3%)	1 (base)	-	1 (base)	-
Suffered headache					
Yes past 2 weeks	28/413 (6.8%)	0.64 (0.26–1.59)	0.331	0.67 (0.23–1.94)	0.456
Yes past year	16/143 (11.2%)	1.55 (0.80–3.00)	0.189 *	1.24 (0.44–3.43)	0.684
No	28/413 (6.8%)	1 (base)	-	1 (base)	-
Chronic heart condition					
Yes	7/35 (20%)	2.31 (0.88–6.10)	0.089 *	1.98 (0.47–8.33)	0.347
No	43/676 (6.4%)	1 (base)	-	1 (base)	-
Chronic liver disease					
Yes	0/3 (0%)				
No	50/708 (7.1%)	1 (base)	-	1 (base)	-
Communal versus private land use ^1^					
Yes	1/45 (2.2%)	0.17 (0.02–1.34)	0.093 *	-	-
No	50/673 (7.4%)	1 (base)	-	-	-
Animals in the house					
Yes	48/650 (7.9%)	0.09 (0.01–1.43)	0.087 *	0.09 (0.01–1.50)	0.093
No	0/52 (0%)	1 (base)	-	1 (base)	-
Access to dam on farm					
Yes	45/563 (8.0%)	2.69 (0.99–7.33)	0.053 *	0.83 (0.30–2.32)	0.718
No	5/141 (3.5%)	1 (base)	-	1 (base)	-
Administration antibiotics new animals					
Yes	2/91 (2.2%)	0.28 (0.07–1.17)	0.080 *	0.27 (0.06–1.15)	0.077
No	49/627 (7.8%)	1 (base)		1 (base)	-
People employed on farm					
0–2	8/187 (4.3%)	1 (base)	-	1 (base)	-
3–5	24/256 (9.4%)	2.88 (1.10–7.50)	0.031 *	2.96 (0.95–9.22)	0.061
6–10	11/134 (8.2%)	2.79 (0.93–8.40)	0.067 *	2.32 (0.70–7.62)	0.166
11–50	7/125 (5.6%)	1.80 (0.56–5.85)	0.324	1.57 (0.35–7.12)	0.556
Sheep or cattle on farm					
No sheep (alone or with cattle/goats)	8/199 (4.0%)	1 (base)	-	1 (base)	-
Yes sheep	5/89 (5.6%)	1.42 (0.46–4.39)	0.538	1.48 (0.47–4.72)	0.502
Yes sheep and cattle	38/430 (8.8%)	2.31 (1.05–5.09)	0.038 *	1.95 (0.88–4.35)	0.100
Goats on the farm					
Yes	7/146 (4.8%)	0.38 (0.14–1.01)	0.051 *	0.37 (0.14–0.99)	0.216
No	44/572 (7.7%)	1 (base)	-	1 (base)	-
Wildlife on the farm					
Yes	25/246 (10.2%)	2.23 (1.18–4.20)	0.014 *	1.84 (0.96–3.55)	0.066
No	26/472 (5.5%)	1 (base)	-	1 (base)	-
Brucellosis on farm past					
Yes	9/84 (10.7%)	1.99 (0.85–4.66)	0.113 *	2.22 (0.89–5.55)	0.087
No	41/627 (6.5%)	1 (base)	-	1 (base)	-
Animals vaccinated against brucellosis					
Yes	24/267 (9.0%)	1.85 (0.98–3.47)	0.056 *	1.77 (0.89–3.52)	0.101
No	26/444 (5.9%)	1 (base)	-	1 (base)	-

^1^ Excluded from the multivariable model due to insufficient response counts.

**Table 2:** Risk factors examined for association with *Brucella* IgG seropositivity via univariable and multiple variable analysis among participants in the veterinary profession within the Free State and Northern Cape provinces, 2015–2016.

Veterinary Professionals		Univariate Analysis		Multivariable Analysis	
Variables	*Brucella* IgG Seropositive n/N (%)	Odds Ratio (CI 95%)	*p*-Value (* < 0.2)	Penalized Odds Ratio (CI 95%)	*p*-Value (** < 0.05)
Sex					
Female	2/70 (2.9%)	1 (base)	-	1 (base)	
Male	14/68 (20.6%)	8.60 (1.83–40.4)	0.007 *	4.45 (1.00–19.89)	0.05 **
Population group					
Coloured or Black African	0/36 (0%)	1 (base)	-	1 (base)	
White	16/102 (15.7%)	11.48 (0.65–203)	0.096 *	46.0 (0.07–30545)	0.248
Age (years)					
22–29	1/28 (3.6%)	1 (base)	-	1 (base)	
30–39	2/50 (4.0%)	1.35 (0.11–16.58)	0.812	1.07 (0.22–5.21)	0.933
40–49	3/20 (15.0%)	4.95 (0.44–55.18)	0.191 *		
50–59	3/20 (15.0%)	5.37 (0.46–63.14)	0.179 *		
≥60	4/9 (44.4%)	24.3 (1.87–315)	0.015 *		
Working as animal healthcare worker (years)					
≤5	1/38 (2.6%)	1 (base)	-	1 (base)	
6–10	1/24 (4.2%)	1.62 (0.85–30.6)	0.747	1.82 (1.21–2.72)	0.004 **
11–20	2/23 (8.7%)	3.29 (0.28–39.2)	0.343		
>20	12/37 (32.4%)	18.54 (2.01–171)	0.011 *		
Job description					
Animal health technician	3/37 (8.1%)	1 (base)			
Veterinarian	13/66 (19.7%)	0.91 (0.65–1.27)	0.584	0.85 (0.53–1.38)	0.513
Other (incl. vet nurse, researcher, wildlife capturers)	0/19 (0%)				
Assisting with birthing of animals ^1^					
Yes	16/98 (16.3%)	15.6 (0.86–282)	0.063 *	-	-
No	0/40 (0%)	1 (base)		-	-
Touching of aborted foetus					
Yes	16/106 (15.1%)	10.6 (0.58–192)	0.111 *	10.2 (0.40–264)	0.160
No	0/32 (0%)	1 (base)	-	1 (base)	
Assisting with surgery					
Yes	16/106 (15.1%)	10.6 (0.58–192)	0.111 *	0.32 (0.00–53.2)	0.661
No	0/32 (0%)	1 (base)		1 (base)	
Working with hoofed animals					
Half to whole day	13/71 (18.3%)	5.08 (1.33–19.42)	0.018 *	4.85 (1.27–18.52)	0.021 **
<1 h	3/67 (4.5%)	1 (base)	-	1 (base)	
Wash hands after touching animals					
Always	15/103 (14.6%)	4.45 (0.80–24.63)	0.088 *	1.62 (0.06–41.7)	0.772
Sometimes	1/33 (3.0%)				
Rarely	0/2 (0%)	1 (base)	-	1 (base)	-
Contact with *Brucella*-positive animals in the past					
Yes	16/76 (21.1)	24.5 (1.43–418)	0.027 *	14.1 (0.77–258)	0.075
No	0/46 (0%)	1 (base)	-	1 (base)	
Suffered needlestick					
Yes	14/90 (15.6%)	2.73 (0.57–13.0)	0.205 *	0.04 (0.00–1.50)	0.078
No	2/48 (4.2%)	1 (base)		1 (base)	
Chronic heart disease					
Yes	4/13 (30.8%)	3.67 (0.91–14.8)	0.067 *	0.04 (0.00–33.1)	0.344
No	12/125 (9.6%)	1 (base)		1 (base)	
Chronic diabetes					
Yes	3/6 (50.0%)	8.0 (1.63–393)	0.011 *	110 (0.16–76656)	0.159
No	13/132 (9.8%)	1 (base)	-	1 (base)	-
No chronic condition					
Yes	11/116 (9.5%)	0.36 (0.11–1.16)	0.085 *	0.49 (0.00–335)	0.829
No	5/22 (22.7%)	1 (base)		1 (base)	

^1^ Excluded from the multivariable model due to insufficient response counts.

## Data Availability

The data presented in this study are available on request from the corresponding author due to privacy and ethical restrictions. The dataset includes geographic coordinates that could identify individual farms and landowners, and therefore cannot be publicly shared. However, anonymized and stratified data that do not contain identifying information can be made available upon reasonable request.

## Supplementary Materials

The following supporting information can be downloaded at: https://www.mdpi.com/article/10.3390/tropicalmed10110302/s1, Figure S1: Reported *Brucella abortus* outbreaks in animals from 1 January 2010 to 31 December 2014 in South Africa. Image courtesy of the Sub-Directorate: Epidemiology of the Directorate Animal Health, Department of Agriculture, Forestry and Fisheries (DAFF).

## Abbreviations

The following abbreviations are used in this manuscript:

aOR	Adjusted odds ratio
CI	Confidence interval
ELISA	enzyme-linked immunosorbent assay
IgG	Immunoglobulin G
OR	Odds ratio
ODK	Open data kit
RBT	Rose Bengal test
RVF	Rift Valley Fever
