# Supplementary material for: Seroprevalence and Associated Risk Factors of Human Brucellosis in a Farming and Animal Health Community in South Africa, 2015–2016
Source: Trop Med Infect Dis. 2025 Oct 23;10(11):302. doi: 10.3390/tropicalmed10110302 (PMC12656221; doi:10.3390/tropicalmed10110302)
Supplement: Supplementary file 1 [file tropicalmed-10-00302-s001.zip › tropicalmed-3884399-supplementary.pdf]

**Supplementary material**

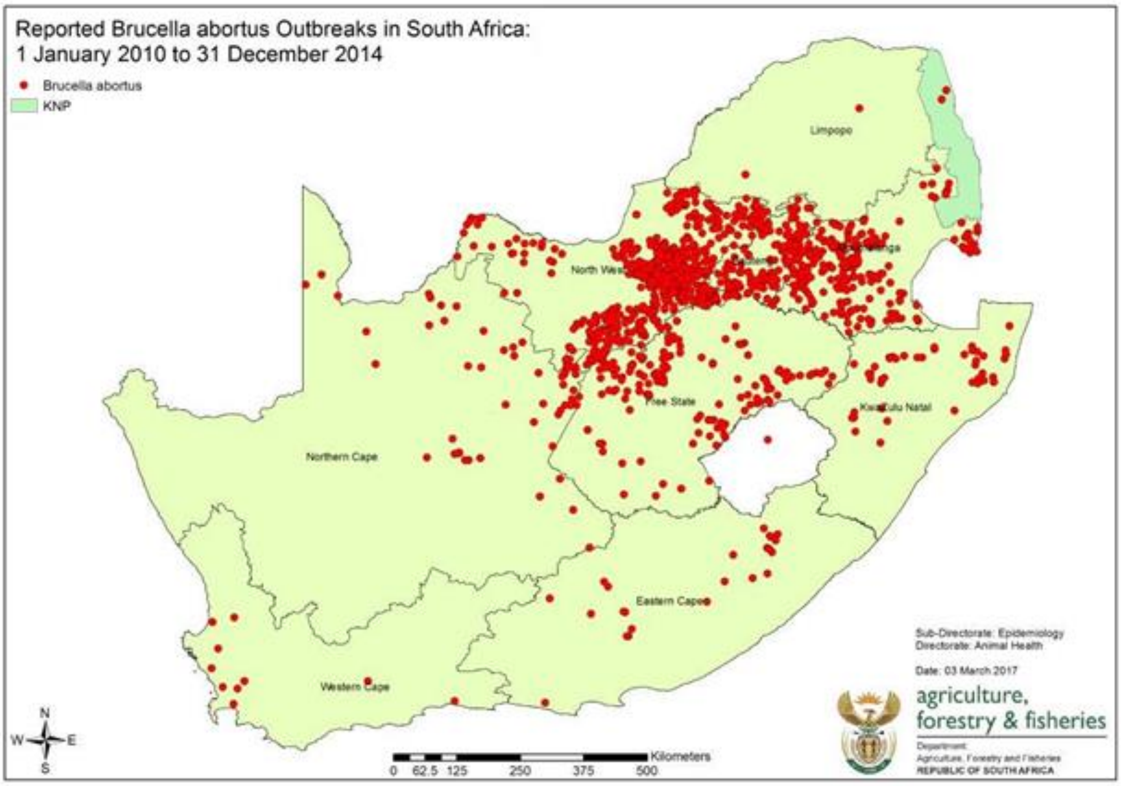


Figure S1: Reported *Brucella abortus* outbreaks in animals from 1 January 2010 to 31 December 2014 in South Africa. Image courtesy of the Sub-Directorate: Epidemiology of the Directorate Animal Health, Department of Agriculture, Forestry and Fisheries (DAFF).
